# Injectable liposomal formulations of opiorphin as a new therapeutic strategy in pain management

**DOI:** 10.4155/fso.14.3

**Published:** 2015-11-01

**Authors:** Natascia Mennini, Paola Mura, Cristina Nativi, Barbara Richichi, Lorenzo Di Cesare Mannelli, Carla Ghelardini

**Affiliations:** 1 Department of Chemistry, University of Florence, Polo Scientifico Sesto Fiorentino, Sesto Fiorentino (FI), Italy; 2 Department of Neurosciences, Psychology, Drug Research & Child Health, University of Florence, Firenze, Italy

**Keywords:** conventional liposomes, improved antinociception, opiorphin, PEGylated liposomes, tail-flick latency test

## Abstract

**Background:**

Conventional and PEGylated liposomes were developed, aimed at improving the pain-killing effect of opiorphin.

**Methods:**

The antinociceptive action of the formulations was investigated on rats (tail-flick test), and compared with that of opiorphin and morphine aqueous solutions (all at 5 mg/kg).

**Results:**

Opiorphin loading in conventional liposomes enabled a 28% AUC increase with respect to free peptide. PEGylated liposomes provided AUC values 80, 60 and 40% higher than free peptide, morphine and opiorphin-loaded conventional liposomes, respectively. Moreover, opiorphin entrapment in PEGylated liposomes increased analgesic effect duration by more than 50%. These results were attributed to the greater effectiveness of PEGylated liposomes in protecting the drug and prolonging its circulation time.

**Conclusion:**

Opiorphin-loaded PEGylated-liposomes can represent a valid alternative to morphine in pain management.

## Abstract (lay abstract)

Opiorphin is an endogenous peptide with a strong analgesic effect, superior to that of morphine and without the several side effects of morphine and morphine-like drugs. Conventional and PEGylated liposomes were developed, aimed at protecting the drug from rapid metabolization and improving its therapeutic efficacy. *In vivo* rat experiments proved that PEGylated liposomes were more effective than conventional liposomes, significantly enhancing intensity and duration of the pain-killing effect. Opiorphin-PEGylated liposomes showed a more intense and prolonged pain-killing effect than morphine at the same dosage. Therefore, this new formulation could represent a noticeable improvement in pain management therapy, as a valid morphine alternative.

**Figure 1.**
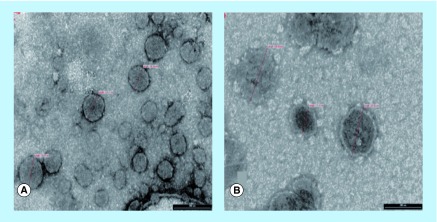
**Transmission electron micrographs of liposomal dispersions.** **(A)** Conventional and **(B)** PEGylated liposomes loaded with opiorphin.

**Figure 2.**
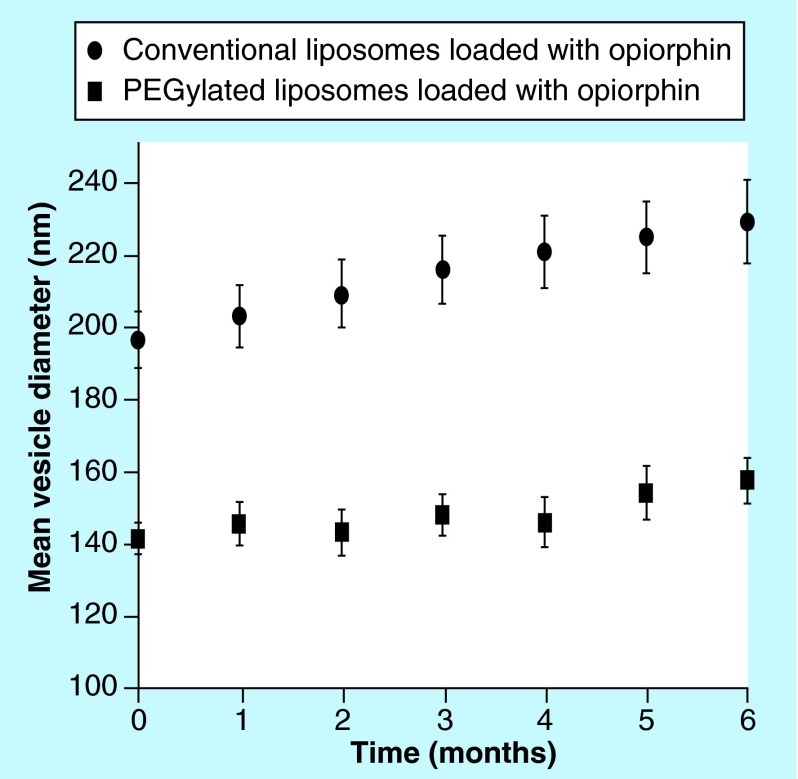
**Mean diameter of vesicles.** Conventional and PEGylated liposomes loaded with opiorphin. Liposomes were stored at 4°C in sealed containers, protected from light.

**Figure 3.**
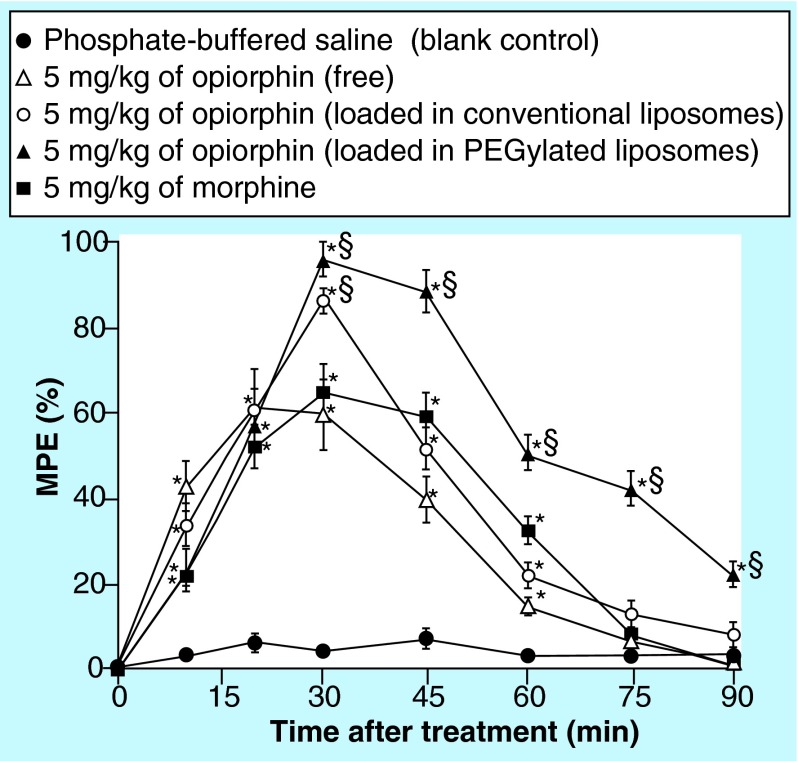
**Normalized mean (±SEM.) degree of antinociception (expressed as percentage of the maximum possible effect) versus time curve achieved by the rat tail-flick latency test.** The presented data were obtained after single-dose intravenous administration of: phosphate-buffered saline (blank control); 5 mg/kg of opiorphin, free or loaded in conventional liposomes or in PEGylated liposomes; 5 mg/kg of morphine. *p < 0.05 vs phosphate-buffered saline treated rats at a given time point. ^§^p < 0.05 vs morphine treatments and/or vs conventional liposomes (n = 6). MPE: Maximum possible effect.

**Figure 4.**
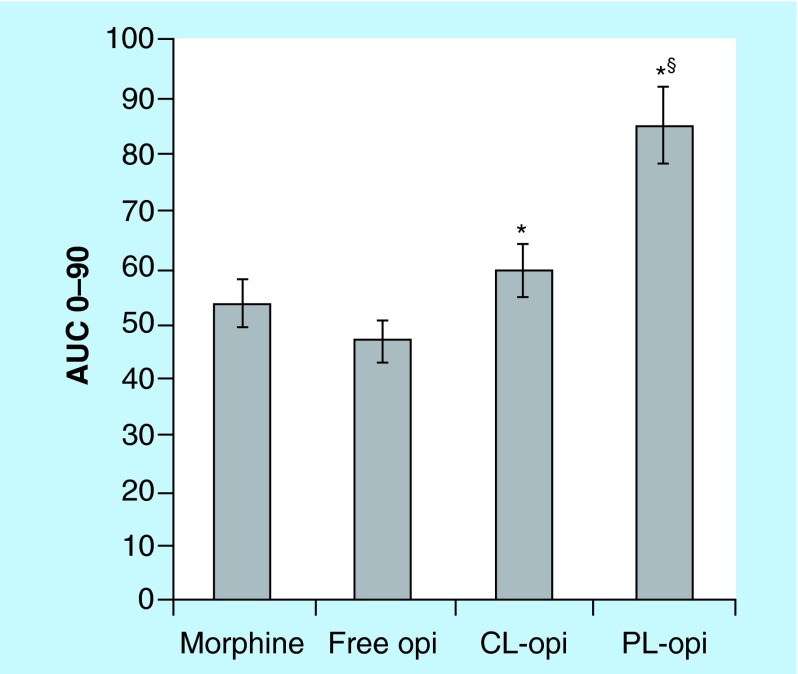
**AUC values after single-dose intravenous administration to rats of 5 mg/kg of different opiorphin formulations or morphine (positive control).** AUC values obtained according to the tail-flick latency test of antinociception (units for AUC values: maximum possible effect[%]/h). Values are mean ±SEM (n = 6). *p < 0.05 vs opi. ^§^p < 0.05 vs morphine, opi and CL-opi. CL-opi: Opiorphin loaded in conventional liposomes; Opi: Free opiorphin; PL-opi: Oopiorphin loaded in PEGylated liposomes.

The treatment of acute and chronic pain, especially in terminally ill patients, is still inadequate, despite the great and extensive efforts made in this regard at both scientific and economic levels. Problems of tolerance, risks of addiction and abuse, appearance of strong side effects (respiratory depression, sedation, reduced gastrointestinal motility) still give rise to considerable restrictions to the use of opioids in the fight against pain. It has been stated that any painkiller agent able to generate fewer side effects than morphine, at equal analgesic doses, would enhance the quality of life of patients [1,2]. The search for new more effective therapeutic treatments against the pain is of fundamental importance not only from an ethical point of view, but also for economic reasons. Numerous studies in fact confirmed the substantial negative economic consequences of the unsuccessful treatment of pain, such as in particular the nonattendance at work of patients and the abuse of anti-inflammatory drugs in the attempt of alleviate their pain. On the contrary, effective treatments to lessen pain symptoms should allow a reduction of the burden of this problem on the society, in terms of both direct and indirect costs [3].

Opiorphin QRFSR-peptide is an endogenous regulator secreted into the human saliva [4], endowed with a strong analgesic effect, even superior to that of morphine [5], and it appears as a promising therapeutic agent in pain management. For instance, administration of opiorphin (1 mg/kg) decreased the paw-licking reflex in rats, inducing the same painkilling effect of 3 mg/kg of morphine, and reduced pain in the ‘pin-pain’ test at a dosage six-times lower than of morphine [6]. The exact mechanism of action of opiorphin is still to be fully clarified, even though a first structure-activity relationship study has been recently performed [7]. It has been demonstrated that this peptide is a powerful dual inhibitor of the encephalin-inactivating metallo-ectoendopeptidases NEP (neutral endopeptidase) and AP-N (aminopeptidase N) [4,7–8]. However, many studies proved that other dual inhibitors of NEP and AP-N encephalinases, such as kelatorphane, induce potent dose-dependent pain-suppression in different animal models of pain, but are unable to produce analgesic effects comparable to those of morphine [9]. On the contrary, opiorphin displayed a more potent analgesic effect than morphine in different pain models. The greater effectiveness of opiorphin with respect to all other peptidase inhibitors suggests that it may produce higher levels of encephalin-like peptides, not only protecting endogenous encephalins from hydrolysis, but also stimulating their release [5]. Another important advantage in the use of opiorphin as painkiller agent is that many of the side effects given by morphine and morphine-like drugs (such as respiratory depression, sedation, constipation, physical and psychic dependence and tolerance) should be absent, since it does not activate directly the opioid receptors and its mechanism of action avoids excessive stimulation of ubiquitously distributed opioid receptors. In fact, subchronic treatments with opiorphin did not develop significant abuse liability and opioid dependence and anti-peristaltic effects were not noted [6,10]. Moreover, repeated treatments with opiorphin, administered intraperitoneally to mice, failed to produce tolerance or morphine-cross-tolerance phenomena [10]. Furthermore, besides its antinociceptive activity, opiorphin showed other beneficial therapeutic effects, particularly interesting from a possible future therapeutic potential perspective. In fact it exhibited antidepressant properties mediated via δ-opioid receptor-dependent pathways, by modulating the concentrations of endogenous encephalins released in response to specific physical and/or psychological stimuli [4,11–13]. However, despite the strong therapeutic potential of opiorphin, its short duration of action, probably due to its rapid degradation by the peptidases in the bloodstream [5,14], represents a serious obstacle to its successful use into clinical practice. In the attempt to overcome this drawback, a structure-activity relationship method was used to design some functional derivatives of opiorphin, which showed improved metabolic stability and unaltered analgesic activity with respect to the natural peptide [14].

An interesting alternative strategy for enhancing opiorphin bioavailability could be the use of a suitable carrier. In fact, different kinds of biodegradable polymeric or lipid-based nanoparticles, micelles, microemulsions, have been proved to be able to allow an effective delivery of a number of therapeutic agents (included analgesic and peptidic drugs), to target sites and protect them from degradation [15–21].

Among these carriers, liposomes offer several advantages with respect to other delivery systems, due to their ability to carry a variety of hydrophobic and hydrophilic molecules (including proteins and peptides), and the biocompatible and biodegradable nature of their components, which makes them suitable also for systemic administration, comprised the intravenous route [22,23]. Liposomes are able to protect the entrapped molecules against enzymatic degradation, allowing for their sustained release by acting as a circulating microreservoir [24–26]. This effect could be very useful in the case of opiorphin, since encapsulation inside the liposome aqueous core should provide a greater resistance to its enzymatic degradation, thus leading to an increase in the peptide bioavailability, and, consequently, analgesic action [10]. However, conventional liposomes (composed of phosphatidylcholine and cholesterol), when administered intravenously, are often rapidly covered by plasma proteins, and this phenomenon can increase their phagocytosis by reticulo-endothelial system cells and reduce their residence time in the blood circulation [27]. A more prolonged blood circulation time of liposomes can be obtained by using poly(ethylene glycol) (PEG) covalently bound to the outer liposome surface, thus obtaining the so called ‘stealth’ liposomes [28]. PEGylation of liposomes generally enables not only the obtainment of a highly hydrated vesicle surface, less prone to interact with plasma proteins, but also the reduction of vesicle aggregation phenomena, thus improving their stability [29].

On the basis of all the above considerations, in this study, an innovative opiorphin formulation was developed by entrapping the peptide in both conventional and ‘stealth’ liposomes, with the aim of protecting it from rapid degradation, after its intravenous administration, thus obtaining a longer analgesic action of the peptide and improving its therapeutic effectiveness.

The obtained colloidal dispersions were characterized in terms of physicochemical, morphological and stability properties, in order to assess their suitability for intravenous administration. The analgesic effect of the developed opiorphin liposomal formulation was evaluated *in vivo* in rats, by the tail-flick test, and compared with that of free opiorphin and morphine.

## Materials & methods

### Materials

Cross-linked lipid polyethylene glycol 2000–distearoylphosphatidylethanolamine (PEG (2000)–DSPE) and cholesterol were purchased from Avanti Polar Lipids Inc. (Alabaster, AL, USA). Egg phosphatidylcholine (PC) was obtained from Fluka (Neu-Ulm, Germany) and stearylamine from Merck (GmBH, Germany). All the amino acid building blocks, the reagents and the resin used for the synthesis of opiorphin were purchased from Bachem AG (Switzerland). All other chemicals and solvents used were of analytical grade.

### Synthesis & characterization of opiorphin

Opiorphin, a five-amino acid polypeptide (Gln-Arg-Phe-Ser-Arg), was synthesized manually by using N^α^-Fmoc solid-phase methodology on preloaded H-Arg(Pbf)-2-chlorotrityl resin (200–400 mesh, loading 0.37 mmol/g, 1 eq, 1 g) [10,30–31]. L-N^α^-Fmoc-protected amino acids (3 equiv) were used. The following amino acid side chain protective groups were used: 2,2,4,6,pentamethyldihydrobenzo-furan-5-sulfonyl (Pbf) for arginine (Arg), *tert*-butyl (*t*-Bu) for serine (Ser), trityl (Trt) for glutamine (Gln). The peptide chain was elongated in consecutive cycles of deprotection and coupling. The coupling reactions were performed at room temperature by 1-hydroxybenzotriazole (HOBt, 3 equiv) and diisopropyl-carbodiimide (DIC, 3 equiv). In the case of Fmoc-Gln(Trt)-OH, coupling was carried out using *O*-(benzotriazol-1-yl)-1,1,3,3-tetramethyluronium tetrafluoroborate (TBTU, 3 equiv) and diisopropyl-ethylamine (DIEA, 6 equiv). Each coupling was performed preactivating the L-N^α^-Fmoc-protected amino acids in dry *N,N*-dimethylformamide (DMF, 0.18 M) into a round bottom flask. Thus, the coupling agents were added to an ice-cooled solution of the L-N^α^-Fmoc-protected amino acids in dry DMF. The reaction mixture was stirred 10 min at 0°C, then warmed at room temperature and stirred 10 min. The preloaded H-Arg-(Pbf)-2-chlorotrityl resin was placed in a glass peptide synthesis column with a frit on the bottom and swollen with dry CH_2_Cl_2_ under N_2_ flow. Each coupling was performed by adding the DMF solution of preactivated L-N^α^-Fmoc-protected amino acids in the peptide synthesis column. Each reaction mixture was mixed 0.5 h under N_2_ flow and 12 h under oscillating agitation. After each coupling the resin was washed with dry DMF (5 × 10 min × 10 ml). The deprotection step of the Fmoc group was carried out using a 25% piperidine solution in dry DMF (2 × 15 min × 18 ml). The deprotected resin was washed with dry DMF (7 × 5 min × 10 ml). Both coupling and deprotection efficiencies were monitored by the bromophenol blue Test. After the peptide assembling, a mixture of (TFA:TIS:H_2_O, 95:2.5:2.5) (1 × 2 h × 9 ml) was used to remove the side chain protective groups and cleave the peptide from the resin. The resin was filtered and the filtrate cooled at -20°C; diethyl ether (60 ml) was then added to precipitate the cleaved peptide. The obtained suspension was stored 18 h at 4°C. The precipitated white solid was separated by centrifugation (3000 rpm × 5 min), washed with diethyl ether, redissolved in water and then lyophilized. The identity and purity of the obtained peptide was determined by HPLC-DAD-ESI MS analysis, using an HP 1100 Liquid chromatograph (Agilent Technologies, CA, USA) equipped with a HP1040 DAD and connected to a mass spectrometer endowed with API-ES (Atmosphere-Pressure-Ionization-ElectroSpray) (Agilent Technologies, CA, USA). The chromatographic analysis was performed using: temperature column 25°C; 10 µl injection volume; mobile phase: a mixture of water added with 0.05% v/v TFA (A) and CH_3_CN added with 0.05% v/v TFA (B). The analysis was carried out under elution gradient (0–9 min, A% [v/v] 95–85; B% [v/v] 5–15). The experimental conditions used for the MS analysis were: N_2_ flow 10 ml/min; atomizer pressure 30 PSI; capillary temperature 250°C; capillary voltage 3500 V; fragmentation energy 60–120 V. The analytical data obtained (m/z 347.25 [M + 2H]^+^, 693.5 [M + H]^+^) corresponded to those of pure opiorphin (molecular weight 692.77). Moreover, the analyses proved that the used synthetic procedure allowed the obtainment of the peptide with a high purity (98%), thus not requiring further costly and time consuming purification procedures, differently from previously described methods [10,31]. The final yield was approximately 60%.

### Preparation of opiorphin-loaded liposomes

Conventional and PEGylated liposomes were prepared according to the thin layer evaporation method, using a previously developed formulation which showed good stability properties [32,33]. Briefly, in the case of opiorphin-loaded conventional liposomes, PC (11 mg/ml), cholesterol (1 mg/ml) and stearylamine (1 mg/ml) were dissolved in 10 ml CHCl_3_ in a 250 ml round-bottomed flask. The organic solvent was then removed under reduced pressure and the evaporation was continued for 2 h, after the dry residue appeared, to completely remove all the solvent. The dry lipid film obtained was hydrated, under mechanical stirring, by addition of 10 ml of physiological solution (NaCl 0.9% w/v), where opiorphin was previously dissolved (1 mg/ml). The hydrated film was then subjected to a water bath heating at 58°C for 5 min and vortexing for 1 min at 30 Hz; the treatment was repeated, performing three cycles in total. In order to reduce the size of the vescicles, the liposomal suspension (2.5 ml) was transferred into a 5 ml round-bottom vial, placed in an ice bath and exposed to probe-sonication (30 Watt, 180 s). The samples were then left to cool down and stored at 4°C in sealed containers under light protection.

Preparation of the PEGylated liposomes differed from that of conventional liposomes only for the addition of PEG (2000)–DSPE (2 mg/ml) to the organic phase.

Preliminary tests allowed for verifying the absence of any degradation phenomena of opiorphin under the experimental conditions used for the vesicles preparation.

### Characterization of conventional & PEGylated liposomes

Average particle size, polydispersity index (PDI) and ζ potential of conventional and PEGylated liposomes were determined by Photon Correlation Spectroscopy (Malvern ZetaSizer Nano ZS90, Malvern, UK) at the set temperature of 25 ± 0.1°C. For these measurements, 20 μl of each liposomal dispersion was diluted 100-folds with NaCl 0.9% w/v, to avoid multiscattering phenomena, and left 30 min at room temperature before analysis. All measurements were performed in triplicate on three different batches (five determinations for each batch). Results were the mean of three different experiments (± SD).

The morphological characteristics of conventional and PEGylated liposomes were examined by Transmission Electron Microscopy (TEM) (Philips CM 12). A drop of liposomal dispersion was placed on a formvar-coated carbon grid and stained by adding a drop of 1% w/w uranyl acetate solution; the excess of staining solution was removed with a filter paper, followed by a through air-drying of the sample.

To determine the drug entrapment efficiency (EE%), loaded liposomes were separated from free opiorphin by size exclusion chromatography, using Sephadex-G50 minicolumns equilibrated with physiological solution (NaCl 0.9% w/v). Each minicolumn was loaded with 50 μl of liposomal dispersion. Purified liposomes were collected by centrifugation of the minicolumns at 1000 rpm for 3 min and then disrupted by adding 200 µl of methanol. After sonication, the sample was transferred into an Eppendorf tube and centrifuged at 12,500 rpm for 5 min. After removing the supernatant, the concentration of opiorphin was determinated in the surnatant by HPLC analysis, as described below, and the entrapment efficiency calculated from the following equation:
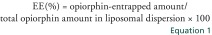



The studies were performed in triplicate.

### HPLC assay of opiorphin

Opiorphin was assayed by HPLC using a Merck Hitachi Elite LaChrom chromatograph (Darmstadt, Germany) equipped with UV-vis detector L-2400 under the following experimental conditions: column Thermo-Scientific™ Hypersil BDS C18 (100 × 4.6 mm, 2.4 µm); injection volume: 20 µl; column temperature: 35°C; flow rate 1 ml/min; mobile phase: water added with 0.05% v/v TFA (A); CH_3_CN added with 0.05% v/v TFA (B). The analysis was performed under this gradient composition: 0–9 min, A% (v/v) 95–85; B% (v/v) 5–15. The retention time of opiorphin under these experimental conditions was 7.6 ± 0.2 min and the LOQ and LOD values were, respectively, 1.14 μg/ml and 0.34 μg/ml.

### Stability studies of liposomal formulations

Stability of conventional and ‘stealth’ liposomes loaded with opiorphin was checked for 6 months. The vesicle dispersions were kept 6 months at 4 ± 1°C in sealed containers under light protection and, at fixed time intervals, examined by mean size and PDI measurements. The entrapment efficiency of the 6 months stored liposomes was also determined.

### *In vivo* studies

The antinociceptive effect of the opiorphin liposomal formulations was assessed by the tail-flick latency test [34]. The test was carried out on adult male Sprague-Dawley rats (200–250 g body weight), provided by Harlan, Italy. The animals were housed in individual cages and placed in the experimental room 24 h before the test, for acclimatization. The animals were kept at 23 ± 1°C with a 12/12 h light/dark cycle, and fed with a standard laboratory diet and free access to water. All the animal experiments were carried out during the light phase, and were performed in agreement with the principles and procedures of both the NIH Guide for the Care and Use of Laboratory Animals of the US National Institutes of Health and the International Association for the Study of Pain. All efforts were made to minimize animal suffering and to limit the number of animals used. The rats, randomly divided into five groups, each of six animals, received single intravenous injections by the tail vein. Each group received one of the following preparations: phosphate-buffered saline (PBS) (blank control group); morphine aqueous solution 5 mg/kg (positive control group); opiorphin aqueous solution 5 mg/kg; liposomal opiorphin 5 mg/kg; PEGylated liposomal opiorphin 5 mg/kg. A constant heat stimulus was applied to the dorsum of the lower third of the rat's tail, and the time required for the animal to withdraw its tail from the heat source, in other words, the tail-flick withdrawal latency, was recorded before and 10, 15, 30, 45, 60, 75 and 90 min after each sample administration. The intensity of the thermal stimulus was adjusted so that the predosing baseline latencies were between 7.0 ± 0.5 s. The baseline latency was obtained for each animal before sample administration as the mean of three consecutive trials. To minimize possible tissue damages to the rats tails, the maximum latency (cut-off time) was established at 17 s. To correct for individual differences in base-line latencies, the antinociceptive data (tail-flick latencies) were normalized, by converting them to percentage of maximum possible effect (%MPE) [10], according to the equation:
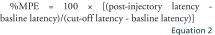



The overall effect of each treatment was also determined as the area under the degree of antinociception versus time curve (AUC), using trapezoidal integration.

The significance of the differences between the antinociceptive effects given by the different treatments was tested using the one-way analysis of variance (ANOVA), followed by Student–Newman–Keuls multiple comparison post-test (GraphPad Prism 4 Software Inc., CA, USA). A p value of < 0.05 was considered to be a significant difference.

## Results

### Physicochemical characterization of opiorphin liposomal formulations

The prepared conventional and PEGylated liposomal formulations of opiorphin were characterized in terms of mean vesicle dimensions, PDI, ζ potential and drug entrapment efficiency (Table 1). The mean diameter of both the opiorphin-loaded liposomal formulations was less than 200 nm; however, the PDI of conventional liposomes was higher than that of the PEGylated ones. The positive ζ-potential value shown by conventional liposomes was due to the presence, into the membrane bilayer, of stearylamine molecules. On the contrary, in the case of PEGylated liposomes, the negatively charged phosphate groups of PEG-DSPE chains counterbalanced to a great extent the positive charges of stearylamine molecules, leading to an almost neutral value of ζ potential. Finally, both kinds of liposomal formulations exhibited a good drug entrapment efficiency, even if it was lower in the case of PEGylated liposomes.

The liposomal vesicles morphology was examined by negative-staining TEM analysis. The obtained TEM images (Figure 1) showed that both conventional and PEGylated liposomal dispersions consisted of vesicles of of discrete and round structures, ranging in size from 150 to 200 nm, which were consistent with the data obtained from particle size measurements (Table 1).

The results of stability studies performed during 6 months on liposomal formulations stored at 4°C in sealed containers protected by light are presented in Figure 2 in terms of mean size of the vesicles as a function of time. Both formulations were substantially stable under the whole storage period, even though in the case of conventional liposomes, a slight but continuous increase in the vesicles dimensions was observed. The entrapment efficiency values after 6 months storage at 4°C were almost unchanged in the case of PEGylated liposomes (67.8 ±2.2 vs 69.2 ± 1.2), while an about 10% decrease was observed for conventional liposomes (81.1 ± 3.6 vs 90.2 ± 1.6).

### *In vivo* studies of the antinociceptive effect of opiorphin liposomal formulations

The effectiveness of the developed liposomal formulations in improving and prolonging the analgesic effect of opiorphin was investigated *in vivo* on rats by the tail-flick latency test, in comparison with the simple aqueous solution of the peptide, all administered by the intravenous route at the dose of 5 mg/kg. An aqueous solution of morphine (5 mg/kg) and a PBS were intravenously administered as positive control and as blank control, respectively. The results of these experiments are presented in Figure 3.

The simple opiorphin aqueous solution produced an evident antinociceptive effect after only 10 min from its administration, which was ever more intense than that given by the morphine aqueous solution after the same interval time. However, as expected, its analgesic action rapidly decreased, becoming similar or even lower than that of morphine, and remaining significantly different from the blank control only for 45 min after injection. When the peptide was administered entrapped in conventional liposomes, a more intense antinociceptive effect was observed in the first 30 min, significantly higher also than that given by morphine (p < 0.05). However, no significant differences were observed after this time with respect to the treatment with the morphine aqueous solution, nor in terms of intensity or duration of action. Moreover, after 60 min its effect was no more significantly different with respect to the blank control (p > 0.05). On the contrary, administration of the PEGylated liposomal formulation of opiorphin allowed a very marked improvement of the extent of the antinociceptive effect, which was significantly more intense than those of both morphine and conventional liposomal opiorphin. Furthermore, it was also clearly more prolonged, remaining significantly higher than the blank control (p < 0.05) until 90 min after the treatment.

These findings were confirmed by the examination of the values of the areas under the degree of antinociception versus time curves (AUC) (Figure 4). The AUC of free opiorphin was lower than that of morphine, probably as a result of the rapid degradation undergone by the peptide in the blood circulation [5]. Protection of opiorphin by entrapment in conventional liposomes allowed an almost 28% increase in the peptide AUC, which became slightly higher (+13%) than that of morphine. However, a very strong improvement of the peptide antinociceptive effect was observed only in the case of opiorphin-loaded PEGylated liposomes, whose AUC value was 80% higher than that of free opiorphin, 60% higher than that of morphine and 40% higher than that of opiorphin-loaded conventional liposomes.

## Discussion

### Physicochemical characterization of opiorphin liposomal formulations

In this work, highly biocompatible opiorphin formulations based on the drug entrapment in liposomal vesicles made up of a mixture of cholesterol, phospholipids and stearylamine, and containing or not PEG chains, have been developed and characterized for physicochemical properties and *in vivo* effects.

The results of Photon Correlation Spectroscopy analysis (Table 1) indicated that both conventional and PEGylated liposomal dispersions had nanometric dimensions, suitable for intravenous administration. The smaller mean particle size presented by PEGylated liposomes could be explained by a steric stabilization effect provided by the PEG chains, that reduced feasible aggregation phenomena among the vesicles. This hypothesis was confirmed by the low value of the PDI of PEGylated liposomes (<0.2), indicative of the formation of a well uniform colloidal dispersion; on the contrary, the conventional liposomes displayed a larger size distribution. It could be hypothesized that the presence, into the membrane bilayer, of the stearylamine molecules, purposely added to stabilize the vesicles by ionic repulsion [32,33] was not enough to completely avoid aggregation phenomena. On the contrary, in the case of PEGylated liposomes, the stability of the liposomal dispersion was assured by the steric hindrance effect of the PEG chains.

The peculiar structure of the liposomes, based on an aqueous core surrounded by a phospholipid shell, allowed the encapsulation of great amounts of opiorphin. The lower drug entrapment efficiency exhibited by PEGylated liposomes with respect to the conventional ones (Table 1), was in agreement with the findings of the study of Nicholas *et al*. [35], where a negative effect of PEGylation on liposome encapsulation efficiency was observed. However, other authors reported an increased entrapment efficiency as a consequence of liposome PEGylation [36,37], or no significant differences in drug entrapment efficiency between conventional and PEGylated liposomes [38,39]. Evidently, different results can be obtained, depending on both the kind of drug to be entrapped and the PEGylation degree of the vesicles.

A frequent instability phenomenon presented by liposomal formulation is the increase in particle size due to aggregation and/or fusion of the vesicles upon storage. An increase in liposomes dimensions generally results in a more rapid uptake by the reticulo-endothelial system, with a consequent faster clearance and shorter half-life. Thus, the obtainment of vesicles able to maintain small and uniform dimensions is of critical importance in the development of a viable pharmaceutical formulation. Stability studies demonstrated that the mean size of PEGylated liposomes remained almost unchanged over 6 months storage at 4°C (Figure 2). In fact, the vesicle size variations were less than 10% with respect to the original values, and also the PDI was stable during time. On the contrary, in the case of conventional liposomes, the observed a progressive slight increase in the vesicles mean size, which after 6 months exceeded the 15% of the initial value, was probably a consequence of some aggregation phenomena. The greater stability of PEGylated liposomes was further confirmed by the absence of appreciable variations of the entrapment efficiency after 6 months storage at 4°C; however, about 10% decrease observed for conventional liposomes can be considered acceptable and indicative of a satisfying stability.

### *In vivo* studies of the antinociceptive effect of opiorphin liposomal formulations

The innovative opiorphin formulation investigated in this paper, based on its entrapment in liposomal vesicles, improved the biopharmaceutical properties of the drug, thus allowing it to exert a pain-killing effect significantly greater than the free drug. This finding does not necessarily imply the ability of the liposomal vesicles to cross the BBB themselves. In fact, it could be also attributed to the improved metabolic stability of the drug, entrapped into the vesicles and/or to the increase of the drug concentration at the luminal surface of BBB cells, which established a blood–brain concentration gradient higher than that reachable after administration of the free drug, thus enhancing its passive diffusion.

In particular, the data obtained from *in vivo* experiments proved that loading of opiorphin in PEGylated liposomes allowed a significant increase in both extent and duration of the peptide analgesic action, with respect not only to the simple opiorphin aqueous solution but also to the opiorphin loaded in conventional liposomes.

The better performance of PEGylated liposomes with respect to the conventional ones can be explained by their peculiar biopharmaceutical properties. In fact, the presence of the PEG chains on their surface provided around the nanocarriers a steric hindrance effect, that on one hand reduced possible vesicle aggregation phenomena [29], improving their stability and effectiveness in protecting the drug from enzymatic degradation, and, on the other, conferred stealth properties to the vesicles [28], prolonging the drug residence time in the hematic circulation. Thus, the combination of these effects enabled for overcoming the drawback of the weak metabolic stability of opiorphin and then for better exploiting its high pain-killing power. In fact, the antinociceptive effect of opiorphin, when loaded in PEGylated liposomes, became significantly more intense and prolonged also in comparison with the morphine solution at the same dosage.

## Future perspective

In this paper, our research group investigated the opportunity of entrapping opiorphin in conventional and PEGylated liposomes, with the aim of improving the peptide therapeutic effectiveness, by reducing the drawbacks related to its rapid enzymatic degradation.

Both the developed formulations showed high drug entrapment efficiency, mean dimensions of the vesicles suitable for intravenous administration, and proved to be substantially stable after 6 months storage at 4°C.

The tail-flick latency test, performed on rats, clearly revealed that PEGylated liposomes were a more efficient carrier for opiorphin with respect to conventional liposomes, allowing for significantly enhancing the extent and duration of the analgesic effect of the peptide, overcoming the limitations to its successful use into clinical practice related to its rapid metabolization [5].

Moreover, entrapment of opiorphin in PEGylated liposomes assured a pain-killing effect higher in intensity and duration also with respect to that of morphine (given at the same dosage), thus representing a noticeable improvement in the therapy for pain management. In fact, the most frequent side-effects associated with the use of morphine or morphine-like drugs, including development of tolerance and physiological dependence, which cause serious complications in common clinical practice, could be eliminated or strongly reduced. This, in turn, would result in a more effective pain management in patients, with a wide modulation of the therapeutic regimens, depending on the pathology characteristics.

In conclusion, the improved stability to enzymatic degradation, the long-circulating properties (conferred by the PEG chains), the consequent possibility of reducing the administration frequency and/or the efficacious dosage of the drug, can be all considered as good reasons to test this new opiorphin formulation in advanced clinical trials.

**Table 1.** **Mean size, polydispersity index, ζ potential and entrapment efficiency values of opiorphin liposomal formulations (mean ± SD, n = 5).**

**Sample**	**Mean size (nm)**	**PDI**	**ζ potential (mV)**	**EE%**
Conventional liposomes	195.7 ± 6.3	0.244 ± 0.015	+14.7 ± 1.4	90.2 ± 1.6
PEGylated liposomes	141.1 ± 4.5	0.184 ± 0.011	-0.62 ± 0.13	69.2 ± 1.2

EE%: Entrapment efficiency; PDI: Polydispersity index.

**Executive summary**

Opiorphin is a natural peptide secreted into the human saliva, endowed with a strong analgesic effect, even superior to that of morphine, and without the several side effects typical of morphine and morphine-like drugs. However, its short duration of action, probably due to its rapid enzymatic degradation, represents a serious obstacle to its successful use into clinical practice.
Conventional and PEGylated liposomes, aimed at protecting the drug from rapid metabolization and improving its therapeutic efficacy, were developed, and both allowed the entrapment of large amounts of the peptide.
*In vivo* experiments in rats proved that PEGylated liposomes were more effective than the conventional ones in significantly enhancing both intensity and duration of the peptide pain-killing effect.
Entrapment of opiorphin in PEGylated liposomes enabled for enforcing and prolonging its pain-killing effect also with respect to morphine, given at the same dosage. Therefore, this new opiorphin formulation can represent a noticeable improvement in the therapy for pain management, as a valid alternative to morphine.
